# Counterfactual Diffusion Modeling Enables Spatially Targeted Reprogramming of Tissue Microenvironments

**DOI:** 10.3390/biology15141097

**Published:** 2026-07-08

**Authors:** Wenhui Ding, Zhenhua Luo, Yuanyan Xiong

**Affiliations:** 1 Department of Pediatric Surgery, The First Affiliated Hospital, Sun Yat-sen University, Guangzhou 510080, China; dingwh6@mail2.sysu.edu.cn; 2 Institute of Precision Medicine, The First Affiliated Hospital, Sun Yat-sen University, Guangzhou 510080, China; 3 Key Laboratory of Gene Engineering of the Ministry of Education, Institute of Healthy Aging Research, School of Life Sciences, Sun Yat-sen University, Guangzhou 510275, China

**Keywords:** generative model, in silico simulation, spatial transcriptomics, spatial proteomics, tumor microenvironment

## Abstract

Spatially resolved single-cell technologies can provide deep insights into cellular heterogeneity and tissue structural characteristics. However, the data obtained are purely observational and cannot reveal the specific mechanisms by which tissues respond to particular perturbations. Most computational models of single-cell perturbations either operate in a non-spatial latent space or fix tissue geometry within a static spatial structure, thereby limiting their ability to integrate molecular profiles with tissue topological remodeling. We propose SPAD-CFR (Spatial Point-cloud Attention-based Diffusion for CounterFactual Reprogramming). Each tissue is treated as a spatial point cloud containing cellular molecular profiles and physical coordinates. We implement Pearl’s three-step workflow for causal inference through deterministic diffusion inversion and sampling. This model can apply interventions to individual cells and generate counterfactual-style tissues in which molecular profiles and spatial coordinates change together. In validation across three datasets, SPAD-CFR reproduces the hierarchical structure of the mouse cerebral cortex, simulates phenotypic distribution differences across different histological grades of breast cancer, and reconstructs hypoxia-associated mesenchymal phenotypes at the invasion margins of triple-negative tumors. In melanoma, activation interventions targeting PD-1+ CD8+ T cells produce spatially confined, distance-dependent bystander cytotoxic effects. Based on these findings, we propose SPAD-CFR, a biologically informed generative framework for conducting counterfactual-style spatial simulations to validate hypotheses regarding microenvironment reprogramming.

## Simple Summary

Predicting the biological behavior of cancer and its response to treatment is difficult because tumors are complex ecosystems in which many different types of cells continuously interact with one another. Traditional tissue samples provide only static snapshots and cannot reveal how the disease alters its physical state. To address this challenge, we have developed a computer model that functions like a virtual laboratory, capable of simulating the changes that may occur when specific conditions within a tumor are altered. Using information from patients with breast and skin cancer, the model was able to reproduce how relatively organized tumor tissues transition into highly abnormal and dangerous forms. It also revealed changes in the movement and behavior of individual cells and predicted the effects of enhancing immune cell function. Most importantly, these simulations can identify which patients are more likely to benefit from specific treatment regimens based on the original distribution of cells within the tissue. This tool enables researchers to rapidly and safely explore various hypothetical scenarios through computation. We believe it will help prioritize the screening of promising therapies and support the development of more precise cancer treatments in the future.

## 1. Introduction

Highly multiplexed spatial transcriptomics and proteomics technologies are now capable of resolving cellular heterogeneity and microenvironmental structures at single-cell resolution [1,2]. However, these measurements are observational in nature and still rely on perturbation experiments [3] to elucidate the causal regulatory mechanisms that determine cell fate and tissue structure formation. Experimental methods have begun to address this goal; for example, spatial Perturb-seq and Perturb-map combine genetic perturbations with spatial observational data [4,5,6]. Such screening methods still face drawbacks such as high cost, difficulty in scaling up, and stringent technical requirements, making it meaningful to develop computational methods capable of simulating perturbation experiments and deriving counterfactual-style inferences.

Various machine learning models have been developed to predict the response of single cells to perturbations. Foundational models such as scGen [7], GEARS [8], CellOT [9], and scGPT [10] can predict changes in gene expression following genetic or chemical perturbations. In recent years, diffusion models have also been applied to the study of perturbations at the single-cell level. For example, the Squidiff model uses a diffusion autoencoder with a semantic latent space to simulate transcriptomic changes during development and drug treatment [11]. These methods all follow the virtual cell paradigm [3], in which each cell is treated as a point in a non-spatial latent space, without explicitly encoding physical spatial relationships. Consequently, extending such models to account for spatially constrained paracrine signaling and the structural topological features of neighboring tissues remains challenging [12].

To leverage spatial information, recent research has increasingly turned to virtual tissue modeling methods, which can predict the local effects of perturbations within an entire tissue. Celcomen developed a mathematically interpretable causal framework that distinguishes between intracellular and intercellular regulation and predicts gene expression following knockout [13]; SpaceTravLR utilizes interpretable convolutional networks to identify spatial microenvironments and simulate how gene knockout reshapes signaling networks in neighboring regions [14]; SpatialProp, meanwhile, employs a graph neural network calibration scheme to propagate the effects of single-cell perturbations throughout the entire microenvironment [15]. More recently, generative and counterfactual approaches have been extended to spatial proteomics, for example to learn perturbations that promote T-cell infiltration into tumours [16] and to map and reprogram human tissue microenvironments [17]. Although these methods have demonstrated the feasibility of modeling spatial perturbations, they essentially rely on static spatial frameworks. Most methods treat physical coordinates as fixed graph structures and propagate deterministic gene perturbations within static grids. However, key biological transformation processes, such as tumor invasion, epithelial–mesenchymal transition (EMT), or immune infiltration, are inherently dynamic processes.

We propose Spatial Point-cloud Attention-based Diffusion for CounterFactual Reprogramming (SPAD-CFR). This algorithm employs a node-level linear attention encoder to assign individual context-aware latent variables to each cell and can directly apply interventions to individual cells in continuous point cloud space. We primarily apply SPAD-CFR to three scenarios: simulating distributional phenotype transitions between breast cancer histological grades; hypoxia-related remodeling phenomena in specific subtypes at the margins of invasive tumors; and spatially confined cytotoxic responses generated by exhausted T cells following activation interventions in melanoma. This algorithm is designed to serve as a computational simulation platform for hypothesis generation, candidate target exploration, and microenvironment reprogramming studies, thereby bridging the gap between single-cell perturbation modeling and spatially resolved tissue simulation.

## 2. Methods

### 2.1. Datasets and Data Preprocessing

We evaluated the generalization capacity and biological fidelity of our framework across three independent, spatially resolved single-cell cohorts spanning both transcriptomic and proteomic modalities.

**MERFISH Mouse Primary Motor Cortex Atlas.** For spatial transcriptomics, we utilized a publicly available MERFISH atlas profiling the mouse primary motor cortex [18]. Specifically, the training set comprised 33 tissue slices derived entirely from a single mouse, whereas the independent testing set consisted of 31 slices obtained from a distinct animal. This configuration guarantees that model evaluation reflects true cross-subject generalization. Physical *y*-coordinates of each tissue slice were geometrically corrected for orientation and min-max normalized to compute a unified cortical depth on a [0,1] scale ( 0.0=pialsurface , 1.0=whitematter ), facilitating topological evaluation across the anatomical layers (L2/3 to L6).

**Imaging Mass Cytometry (IMC) Breast Cancer Cohorts.** We modeled complex tumor microenvironments utilizing high-dimensional IMC datasets characterizing primary invasive breast cancer [19]. The multiplexed panel encompassed over 35 spatially resolved protein markers quantifying tumor lineage, immune infiltration, and stromal components. We isolated pathological microenvironments by strictly excluding patient-matched healthy control tissues and unannotated cells. The analysis leveraged two distinct cohorts. The Basel cohort characterized tumor microenvironments across histological grades. Following quality control, the dataset contained 767,833 single cells across 289 tumor cores. We executed a patient-level stratified split based on histological grade (Grades 1, 2, and 3), allocating 224 patients (230 cores; 600,818 cells) to the training phase and reserving 57 patients (59 cores; 167,015 cells) for the final independent evaluation. The Zurich cohort facilitated the analysis of intra-tumoral spatial heterogeneity, specifically targeting the functional dichotomy between hypoxic central regions and invasive peripheral margins. The processed dataset comprised 359,233 cells. We enforced a strict patient-level partition stratified by major clinical subtypes (Triple Negative, ${\mathrm{HR}}^{+}{\mathrm{/HER2}}^{-}$ , and ${\mathrm{HR}}^{+}{\mathrm{/HER2}}^{+}$ ). The training set included 56 patients (206 cores; 273,730 cells), while the testing set contained 15 patients (58 cores; 85,503 cells). Preserving subtype distributions across the split was critical for preventing bias during the subtype-specific microenvironmental remodeling analysis, thereby ensuring that the test set contained a balanced representation of both central and peripheral topologies across all biological groups.

**Imaging Mass Cytometry Melanoma Cohort.** Targeted in silico immune checkpoint blockade simulations were conducted using a spatial multiparametric IMC dataset of melanoma patients [20]. Initial preprocessing eliminated normal adjacent tissue controls, retaining only bona fide tumor microenvironments. Single-cell raw intensity values were transformed using an inverse hyperbolic sine (arcsinh) function with a cofactor of 1, stabilizing variance while conforming to standard mass cytometry practices. We maintained the strict T cell phenotypic definitions established in the original publication. Specifically, empirical gating thresholds were applied wherein ${\mathrm{CD8}}^{+}$ T cells exhibiting an average arcsinh-transformed expression greater than 1.5 for TCF7 or PD-1 were classified as positive for those respective markers. Consistent with our overarching methodology, dataset partitioning was executed with a rigorous patient-level isolation strategy. For the melanoma cohort, the training partition comprised 55 patients and 119 cores (27 responder and 41 non-responder cores; the remaining cores were not annotated for response), totalling 730,198 cells; and the held-out test partition comprised 14 patients and 40 cores (12 responder and 17 non-responder cores).

**Prevention of Data Leakage.** Across all case studies, models were exposed exclusively to the training partitions. Consequently, all generative interventions, zero-shot concept vector computations ( Δc ), and downstream topological evaluations were derived strictly from unseen testing cohorts. The concept vector ( Δc ) is estimated based on the groups defined in the test queue. This method utilizes the biological metadata associated with the retained tissue samples, but does not expose the generative model to the test data during training, nor does it reference clinical outcome labels. Therefore, we describe this design as one that prevents the model from accessing test data during the training phase, rather than completely eliminating any form of information transfer.

### 2.2. Problem Formulation: A Structural Causal Model for Spatial Biology

We formalize the counterfactual spatial reprogramming framework within the framework of a Structural Causal Model (SCM) [21]. A spatial transcriptomic or proteomic sample is represented as a multidimensional point cloud $X_{0}=\left[F_{0},P_{0}\right]\in R^{N\times (F+2)}$ , where *N* denotes the number of cells, $F_{0}\in R^{N\times F}$ encapsulates the protein/gene expression profiles, and $P_{0}\in R^{N\times 2}$ represents the 2D physical coordinates.

The generative mechanism of the tissue architecture is governed by two unobserved causal factors: (1) an endogenous, node-level structural variable $Z\in R^{N\times d_{\mathrm{node}}}$ that defines the intrinsic biological state and local microenvironmental context of each cell, and (2) an exogenous noise variable ϵ∼N(0,I) that captures stochastic spatial perturbations. The SPAD-CFR architecture is designed to follow the three-step procedural semantics of counterfactual inference proposed by Pearl [21]: abduction (inferring the exogenous noise and endogenous states from observations), action (applying targeted spatial interventions to Z ), and prediction (generating the counterfactual tissue state via the deterministic reverse diffusion process, which corresponds to the probability flow ordinary differential equation [22,23]).

To clarify the scope of causal explanations, we first outline the underlying structural assumptions. We propose a generative process X=g(Z,ϵ) , which consists of an endogenous node-level state *Z* and an exogenous variable ϵ∼N(0,I) . We interpret abduction as the reconstruction of a latent representation consistent with the observed outcomes, an action as a modification of *Z*, and a prediction as a deterministic re-generation. For this framework to align with Pearl’s concept of counterfactual inference, two premises must be satisfied: (i) *Z* must represent the biologically meaningful endogenous state of each cell; and (ii) ϵ must be an exogenous variable that is statistically independent of the intervened state. Neither of these premises can be verified solely through observational spatial data, and while DDIM inversion can establish a reproducible mathematical mapping to the latent encoding, it cannot in itself prove that this encoding represents the true exogenous noise in the causal model. Therefore, we do not claim formal causal identifiability. SPAD-CFR is more accurately described as a biologically informed conditional generation framework, whose inversion–intervention–sampling process adheres to counterfactual semantics. Throughout this paper, we use the term “counterfactual” in its practical sense, meaning that the generated tissues should be regarded as a simulation result based on the intervention conditions. Its core value lies in generating testable hypotheses, rather than directly asserting definitive causal effects.

### 2.3. Model Architecture: SPAD-CFR

The SPAD-CFR system consists of a joint network parameterized by two core components: a node-level conditional encoder and a conditional denoising diffusion network.

**Node-level Conditional Encoder.** To capture the independent state and spatial context of each cell, the encoder $E_{\theta }$ maps the original point cloud $X_{0}$ into the latent space. We employed two independent Multi-Layer Perceptrons (MLPs) to process the expression $F_{0}$ and coordinates $P_{0}$ into uniform hidden dimensions, which are subsequently concatenated. To circumvent the $O(N^{2})$ computational bottleneck of standard self-attention, we utilized a Linear Attention Transformer mechanism [24], which reduces complexity to $O(N\cdot d_{\mathrm{hidden}})$ and enables efficient global context aggregation for tissue slices encompassing thousands of cells. The encoder employs GELU activations [25] throughout, which provide $C^{\infty }$ smoothness. This smoothness provides the continuous differentiability well suited to stable integration of the probability-flow ODE during inversion and sampling. The encoder yields the condition matrix $Z=\{z_{1},z_{2},\ldots ,z_{N}\}$ , where each $z_{i}$ encodes the localized identity of cell *i*.

**Conditional Denoising Network.** The reverse generative process is parameterized by $D_{\phi }\left(X_{t},Z,t\right)$ , designed to predict the noise-free point cloud ${\hat{X}}_{0}$ conditioned on the perturbed state $X_{t}$ , the normalized continuous diffusion timestep *t*, and the latent condition Z . Feature fusion is executed by independently projecting $X_{t}$ , time embeddings, and Z via MLPs, followed by element-wise addition:

$$H={\mathrm{MLP}}_{\mathrm{in}}\left(X_{t}\right)+{\mathrm{MLP}}_{\mathrm{time}}\left(t\right)+{\mathrm{MLP}}_{\mathrm{cond}}\left(Z\right)$$

The fused representation H is then processed by a multi-layer Linear Attention Transformer [24]. Finally, two independent linear projection heads separately decode the denoised molecular expressions ${\hat{F}}_{0}$ and physical coordinates ${\hat{P}}_{0}$ . The full architecture, training, and evaluation hyperparameters are provided in Table S1.

### 2.4. Forward Diffusion and Joint Training Objective

Unlike standard tabular diffusions, our model simultaneously and independently corrupts both molecular expressions and spatial coordinates. Following a cosine noise schedule [26], the noisy joint point cloud at timestep t∈(0,T] is constructed by applying shared diffusion coefficients to each modality with independently sampled noise:

$$F_{t}=\sqrt{{\bar{\alpha }}_{t}}\;F_{0}+\sqrt{1-{\bar{\alpha }}_{t}}\;\epsilon_{f},\;P_{t}=\sqrt{{\bar{\alpha }}_{t}}\;P_{0}+\sqrt{1-{\bar{\alpha }}_{t}}\;\epsilon_{p}$$

where $\epsilon_{f},\epsilon_{p}∼N\left(0,I\right)$ are independently sampled noise matrices. The spatial coordinates P and the molecular features F share an identical diffusion timestep *t* and its corresponding coefficient ${\bar{\alpha }}_{t}$ , ensuring temporal synchronization of the two modalities while precluding spurious cross-modal noise correlation.

The encoder $E_{\theta }$ and the denoising network $D_{\phi }$ are trained jointly end-to-end without isolated pre-training. To rigorously avoid spurious gradient contributions from zero-padded entries under variable-length inputs, both the molecular expression and structural position losses are strictly evaluated only on the subset of valid cells. Let *V* denote the index set of valid (unpadded) cells within a given tissue core. The overall objective function is formulated as a weighted sum:

$$L_{\mathrm{total}}=L_{\mathrm{feat}}+\lambda \;L_{\mathrm{pos}}$$

where λ is a balancing hyperparameter that scales the magnitude of the structural gradients to align with the feature loss.

The feature loss minimizes the Mean Squared Error (MSE) of the predicted protein profiles, evaluated exclusively over the valid cells:

$$L_{\mathrm{feat}}=E_{X_{0},\;\epsilon_{f},\;t}\left[E_{i\in V}\left[{∥{\hat{f}}_{i}-f_{i}∥}_{2}^{2}\right]\right]$$

where ${\hat{f}}_{i}\in R^{F}$ and $f_{i}\in R^{F}$ represent the predicted and ground-truth molecular feature vectors of cell *i*, respectively.

Directly minimizing the absolute coordinate MSE is highly sub-optimal due to the lack of translation and rotation invariance in spatial geometries. Following the pairwise distance preservation strategy introduced in LUNA [27], we enforced topological consistency by calculating the MSE between the pairwise Euclidean distance matrices of the predicted and ground-truth coordinates. By formulating this objective as an expectation over all nested pairs of valid cells, the structural position loss is defined as:

$$L_{\mathrm{pos}}=E_{X_{0},\;\epsilon_{p},\;t}\left[E_{i,j\in V}\left[{\left(∥{\hat{p}}_{i}-{\hat{p}}_{j}{∥}_{2}-{∥p_{i}-p_{j}∥}_{2}\right)}^{2}\right]\right]$$

where ${\hat{p}}_{i},{\hat{p}}_{j}\in R^{2}$ and $p_{i},p_{j}\in R^{2}$ denote the predicted and actual 2D physical coordinates of cells *i* and *j*.

Although the pairwise distance matrix scales quadratically in the number of valid cells, it is computed once per sample locally during the loss evaluation phase and does not participate in the deep sequential attention stack, thereby maintaining the overall scalability of the architecture.

### 2.5. Deterministic DDIM Inversion and Sampling

To rigorously execute the Abduction and Prediction phases of our Structural Causal Model, we employ the deterministic framework of Denoising Diffusion Implicit Models (DDIM) [22]. In a generalized diffusion formulation, transition trajectories incorporate a stochastic variance parameter ( $\sigma_{t}$ ) that controls random noise injection. By explicitly setting this variance to zero ( $\sigma_{t}=0$ ), the underlying Stochastic Differential Equation (SDE) degenerates into a probability flow Ordinary Differential Equation (ODE) [22,23]. The deterministic probability-flow ODE defines an invertible correspondence between an observed point cloud $X_{0}$ and its latent representation $X_{T}$ . In practice we integrate this trajectory with a finite number of discrete steps, so the mapping we use is a close numerical approximation to this correspondence rather than an exact inverse.

**Abduction via DDIM Inversion.** During the SCM Abduction phase, we infer the exogenous latent representation associated with the observed tissue. We achieve this by integrating the generative ODE backward in time (i.e., transitioning from a cleaner state *s* to a noisier state *t*, where t>s ). At each step, the network predicts the uncorrupted origin ${\hat{X}}_{0}=D_{\phi }\left(X_{s},Z,s\right)$ conditioned on the current state and the original latent condition Z . The subsequent noisier state $X_{t}$ is deterministically computed as:

$$X_{t}=\sqrt{{\bar{\alpha }}_{t}}\;{\hat{X}}_{0}+\sqrt{1-{\bar{\alpha }}_{t}}\;\frac{X_{s}-\sqrt{{\bar{\alpha }}_{s}}\;{\hat{X}}_{0}}{\sqrt{1-{\bar{\alpha }}_{s}}}$$

This deterministic forward trajectory uniquely maps the observed point cloud $X_{0}$ to its corresponding latent noise representation $X_{t^{\prime }}$ . To assess the contribution of this inversion step, we compared it against random Gaussian initialization under otherwise identical conditioning. The intervention remained confined to the targeted cells under either initialization, with inversion providing a small gain in non-targeted expression fidelity (Supplementary Figure S3).

**Prediction via DDIM Sampling.** During the Prediction phase, after applying the targeted spatial perturbation to obtain the modified latent condition $Z^{\prime }$ , we simulate the counterfactual tissue by integrating the same deterministic ODE forward in time (transitioning from *t* to *s*, where s<t ). By sharing an identical ODE trajectory, inversion and sampling enforce the Minimal Change Principle [21] on the latent structural equations. Crucially, the unperturbed latent conditions and the inferred exogenous representation are carried through largely unchanged, the observable point cloud is dynamically reconstructed via linear attention. This formulation models localized biophysical interactions without causing unwarranted global background corruption.

### 2.6. In Situ Concept Extraction and Counterfactual Reprogramming

**Concept Discovery.** A defining hallmark of tumor microenvironments is their profound intra- and inter-patient heterogeneity. To accurately capture batch-specific nuances and the in situ biological context of the target phenotype, the structural concept vectors ( Δc ) are derived directly from the evaluation cohort. Estimating concept vectors from the training partition introduces inter-patient variability arising from strictly patient-disjoint data splits, which degrades the specificity of the resulting concept axis. By contrast, deriving Δc from the evaluation cohort using only zero-shot biological gating (e.g., canonical marker thresholds) with no reference to clinical outcome labels does not constitute label leakage and preserves the biological fidelity of the extracted concept. For a given biological concept, we aggregated the latent representations of the conceptually positive ( $S_{\mathrm{target}}$ ) and negative ( $S_{\mathrm{source}}$ ) populations. The concept axis was computed as the difference in their empirical centroids:

$$\Delta c=\frac{1}{|S_{\mathrm{target}}|}\sum_{i\in S_{\mathrm{target}}}z_{i}-\frac{1}{|S_{\mathrm{source}}|}\sum_{j\in S_{\mathrm{source}}}z_{j}$$

To confirm that these effects depend on the specific biological direction rather than on the act of perturbation, we compared the biological Δc against norm-matched random and dimension-shuffled vectors. Only the biological direction reproduced the effector induction and the downstream spatial bystander response (Figure S4).

**Targeted Spatial Intervention.** Executing the SCM requires instantiating the exogenous spatial noise (Abduction), modifying the endogenous latent state (Action), and simulating the outcome (Prediction). During the Action phase, an intervention scalar α∈R dictates the perturbation intensity. A spatial indicator subset $S_{\mathrm{perturb}}$ (e.g., exclusively tumor cells) is defined, and the latent condition is selectively updated:

$$z_{i}^{\prime }=\left\{\begin{array}{cc} z_{i}+\alpha \cdot \Delta c, & \mathrm{if}\;i\in S_{\mathrm{perturb}}\\ z_{i}, & \mathrm{otherwise} \end{array}\right.$$

To generate the counterfactual point cloud $X_{\mathrm{cf}}$ , we first obtain the exact exogenous noise representation $X_{t^{\prime }}$ of the observed point cloud $X_{0}$ via deterministic DDIM inversion (as detailed in the previous subsection). We then initialize the diffusion model from this inverted state $X_{t^{\prime }}$ conditioned on the perturbed latent matrix $Z^{\prime }=\left\{z_{1}^{\prime },\ldots ,z_{N}^{\prime }\right\}$ and execute the deterministic DDIM sampling process.

This approach accurately translates the localized single-cell perturbations into emergent downstream molecular and microenvironmental spatial remodeling.

### 2.7. Quantitative Evaluation Metrics

To assess the topological, microenvironmental, and clinical fidelity of the counterfactual simulations, we deployed a suite of spatial statistics and machine learning evaluations. All metrics were computed exclusively on the independent test cohorts to ensure out-of-distribution generalizability.

**Spatial Autocorrelation.** To quantify the macroscopic functional clustering of the simulated tissue, we computed global Moran’s *I* [28] for continuous marker expressions. The spatial weight matrix W was constructed using a *k*-nearest neighbors (*k*-NN) graph ( k=15 ). To adjust for varying local cellular densities across the tissue point cloud, the matrix was row-standardized such that the weights of all neighbors for a given cell sum to 1. Given the row-standardized W , global Moran’s *I* is formally defined as:

$$I=\frac{\sum_{i=1}^{N}\sum_{j=1}^{N}w_{ij}\left(x_{i}-\bar{x}\right)\left(x_{j}-\bar{x}\right)}{\sum_{i=1}^{N}{\left(x_{i}-\bar{x}\right)}^{2}}$$

where *N* is the total number of cells, $x_{i}$ denotes the molecular expression of cell *i*, $\bar{x}$ is the mean expression across all cells, and $w_{ij}$ is the spatial weight between cells *i* and *j*.

**Spatial Point Pattern Analysis.** The physical dispersion and invasive aggregation of tumor cells were evaluated using Besag’s *L*-function [29], a variance-stabilized transformation of Ripley’s *K*-function: $\hat{L}\left(r\right)=\sqrt{\hat{K}\left(r\right)/\pi }$ . We utilized the implementation provided by Squidpy [30], estimating $\hat{K}\left(r\right)$ across the coordinate space via the empirical spatial intensity $\hat{\lambda }=N/A$ , where *N* represents the total number of tumor cells and *A* is the estimated convex hull area of the tissue core. No explicit edge correction was applied to Ripley’s *L* estimator; identical coordinate handling, radius ranges, and procedures were used for all groups, and conclusions are drawn from between-group differences rather than the absolute L(r) of any single core. The metric L(r)−r was reported across sweeping search radii *r*. Values greater than zero indicate spatial clustering (homotypic nesting), whereas values approaching zero denote invasive dispersion corresponding to a Poisson point process (Complete Spatial Randomness, CSR).

**Neighborhood Enrichment and Interaction Dynamics.** To capture topological rewiring within the microenvironment, we quantified heterotypic and homotypic cell–cell interactions using a permutation-based neighborhood enrichment analysis. For each interacting cell-type pair (*i*, *j*), the observed co-occurrence frequency ( $E_{ij}$ ) was computed across the spatial nearest-neighbor graph. To establish a rigorous null hypothesis of random spatial mixing while strictly preserving the underlying physical geometry of the tissue, we performed 1000 independent random permutations of the categorical cell phenotype labels across the fixed graph nodes. The interaction magnitude was subsequently quantified as a *Z*-score:

$$Z_{ij}=\frac{E_{ij}-\mu_{ij}}{\sigma_{ij}}$$

where $\mu_{ij}$ and $\sigma_{ij}$ are the mean and standard deviation of the interaction frequencies derived from the 1000 empirical permutations. Positive scores ( $Z_{ij}>0$ ) denote spatial co-localization (attraction), while negative scores denote spatial segregation (avoidance). Reported *p*-values for the differential interaction analysis are sample-level and uncorrected for multiple comparisons; the corresponding panels are intended as a descriptive comparison of interaction structure rather than as a set of individually significance-tested discoveries.

**Local Phenotypic Entropy.** Tumor cells in the TNBC cohort were clustered into phenotypic meta-states by *k*-means on 14 functional markers. We set K=5 as the smallest value that separated the canonical TNBC archetypes (quiescent, hypoxic, proliferative, mesenchymal, and hyper-mitotic) without redundant sub-clusters. To quantify the ecological complexity of the invasive margin in the TNBC simulation, we evaluated the spatial Shannon entropy. To isolate intrinsic tumor heterogeneity from generic stromal infiltration, the KD-tree spatial query was restricted exclusively to neighboring tumor cells. For each focal tumor cell *i*, we extracted its local microenvironment defined by its k=20 nearest tumor neighbors (yielding 21 cells inclusive of the focal node). The local phenotypic entropy $H_{i}$ (in bits) was computed over the frequency distribution of the M=5 discrete functional meta-states C :

$$H_{i}=-\sum_{c=1}^{M}p_{i,c}\log_{2}p_{i,c}$$

where $p_{i,c}$ represents the proportion of meta-state *c* within the focal cell’s local 21-cell neighborhood. High entropy values indicate a highly admixed, heterogeneous tumor sub-clone distribution, whereas values approaching zero reflect homotypic structural dominance.

**Classifier-Based Realism Assessment.** To holistically evaluate whether the generated single-cell profiles authentically recapitulate clinical phenotypes, we executed a classifier-based realism assessment using an independent Random Forest classifier. To capture both autonomous cell states and non-autonomous environmental context, the input feature space for each cell was constructed by concatenating its intrinsic protein expression profile with the localized mean and standard deviation of the expressions of its k=10 nearest spatial neighbors, yielding a 99-dimensional spatio-molecular signature. The classifier was trained exclusively on the real, independent training cohort to distinguish bona fide Grade 1 from Grade 3 malignancies. To determine the classification threshold without introducing selection bias on the training labels, we leveraged the out-of-bag (OOB) predicted probabilities generated intrinsically during the Random Forest bootstrap procedure [31]. The optimal decision threshold was identified by maximizing Youden’s *J* statistic [32] ( J=Sensitivity+Specificity−1 ) over the OOB probability scores of the training cohort. The pre-trained model was then deployed as a blinded judge on the counterfactual target cells generated from the testing cohort; cells whose predicted probability of Grade 3 malignancy exceeded this OOB-derived threshold were deemed successfully reprogrammed. The resulting conversion rate served as an unbiased, multi-dimensional metric for evaluating the success of the simulated phenotypic reprogramming.

## 3. Results

### 3.1. A Structural Causal Diffusion Framework for Spatial Counterfactual Inference

**Figure 1:**
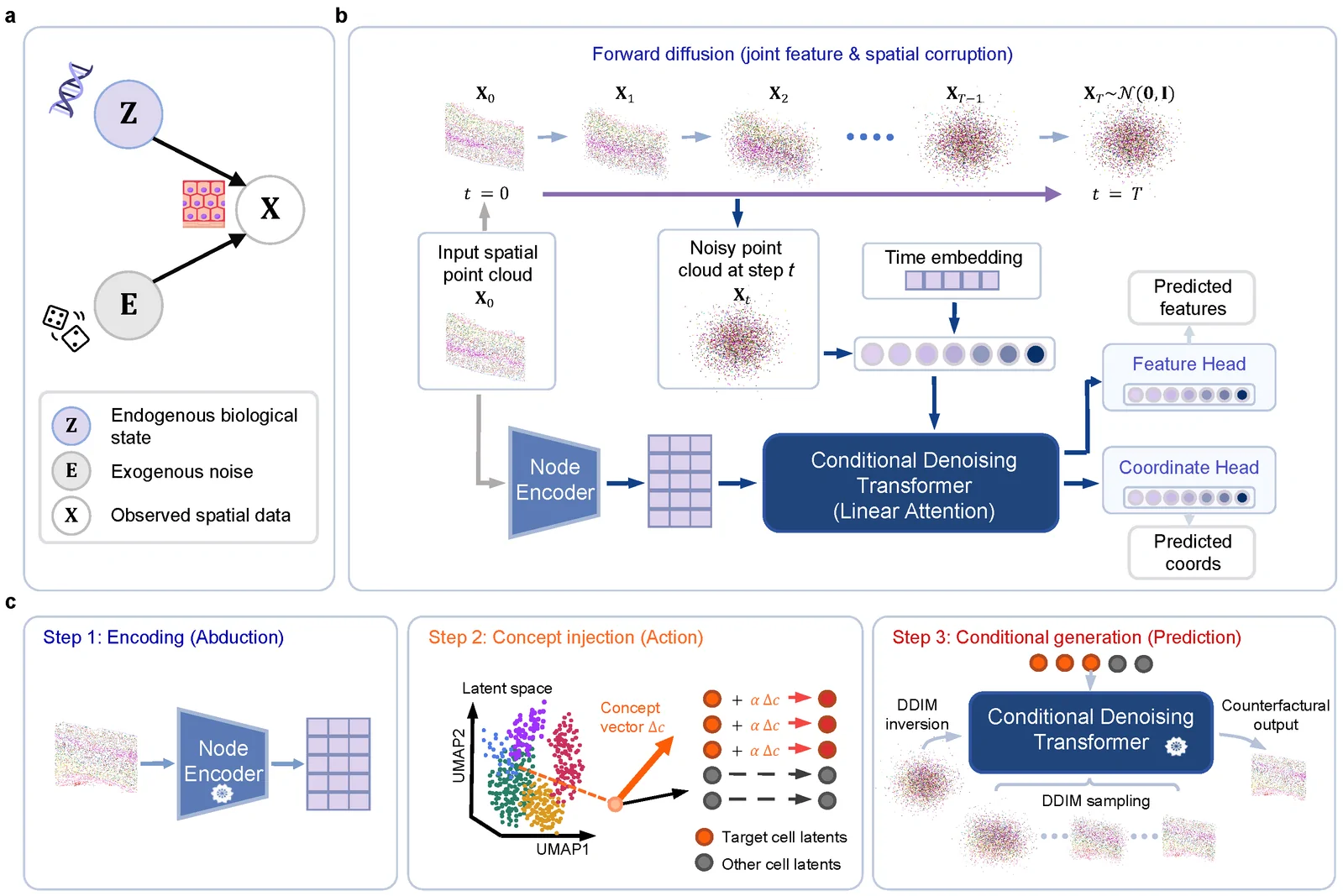
**Overview of the SPAD-CFR.** (**a**) The causal graph used for tissue generation: Observed point cloud data (*X*) are considered to originate from endogenous node-level cellular states (*Z*) and exogenous spatial noise variables (*E*). (**b**) Model architecture: A node encoder maps each input cell to endogenous latent variables, which are then combined with a diffusion time embedding and used as conditions for a denoising transformer with a linear attention mechanism. Two output heads reconstruct molecular features and 2D coordinates from the jointly corrupted point cloud. The ellipsis (⋯) denotes intermediate diffusion timesteps omitted for clarity. (**c**) Counterfactual-style workflow: The abduction step simultaneously encodes the original cellular states and inverts them to the exogenous latent representation; the action step adds a biological concept vector ( Δc ) only to the endogenous latent states of the target subpopulation; the prediction step regenerates the tissue structure via deterministic sampling, ultimately yielding output results based on the intervention conditions. Colors in the latent space distinguish distinct cell subpopulations; orange and gray circles indicate target and non-target cell latents, respectively, and the concept vector ( Δc ) is added only to the target latents (solid arrows), whereas non-target latents remain unchanged (dashed arrows).

We developed SPAD-CFR, a counterfactual reprogramming framework formalized through a Structural Causal Model (SCM) (Figure 1a) [21]. In this formulation, the observed spatial multi-omics point cloud (*X*) is governed by two distinct causal factors: an endogenous, node-level structural variable (*Z*) capturing the intrinsic identity and microenvironmental context of each cell, and an exogenous background noise variable (*E*) accounting for stochastic spatial arrangements.

To parameterize this causal generative mechanism, SPAD-CFR employs a dual-branch neural architecture integrated with a joint continuous diffusion process (Figure 1b) [33]. Unlike standard tabular diffusion models, our framework simultaneously and independently corrupts both molecular feature profiles and physical 2D coordinates. A node encoder first compresses the uncorrupted input tissue into the continuous latent condition matrix *Z*. Subsequently, a conditional denoising transformer, utilizing linear attention [24] to accommodate point clouds containing thousands of cells, is trained to reverse the joint corruption. Guided by *Z* and temporal embeddings, the transformer predicts the denoised tissue state via two independent linear projection heads, learning the coupled probability distribution of molecular expression and spatial topology.

The operational workflow of SPAD-CFR follows the three-tier semantics of Pearl’s counterfactual inference [21]: abduction, action, and prediction (Figure 1c). During the abduction phase, the model extracts the endogenous condition *Z* while utilizing deterministic Denoising Diffusion Implicit Models (DDIM) inversion [22] to map the observed tissue point cloud $X_{0}$ back to its latent noise representation $X_{T}$ , isolating the exogenous background *E* unique to the observed tissue. Next, in the action phase, biological transition vectors ( Δc ) are identified within the node-level latent space via zero-shot concept discovery. Targeted perturbations are executed by selectively injecting Δc into the latent representations of defined cellular subpopulations, leaving the latent representations of non-targeted cells unchanged. Finally, during the prediction phase, the counterfactual tissue is generated by deterministic DDIM sampling, initialized from the inverted noise $X_{T}$ and conditioned on the modified latent matrix.

Because the deterministic probability flow ordinary differential equation (ODE) [23] enforces Pearl’s Minimal Change Principle [21] at the latent causal level, the exogenous background noise and the intrinsic states of non-targeted cells remain isolated from the primary intervention. However, instead of rigidly freezing unperturbed cells in the observable space, the conditional denoising transformer dynamically propagates the targeted intervention through its spatial attention mechanism. Consequently, SPAD-CFR preserves global tissue architecture while simulating context-aware microenvironmental rewiring, such as contact-dependent bystander effects.

### 3.2. Latent Space Topology and In Silico Spatial Reprogramming

**Figure 2:**
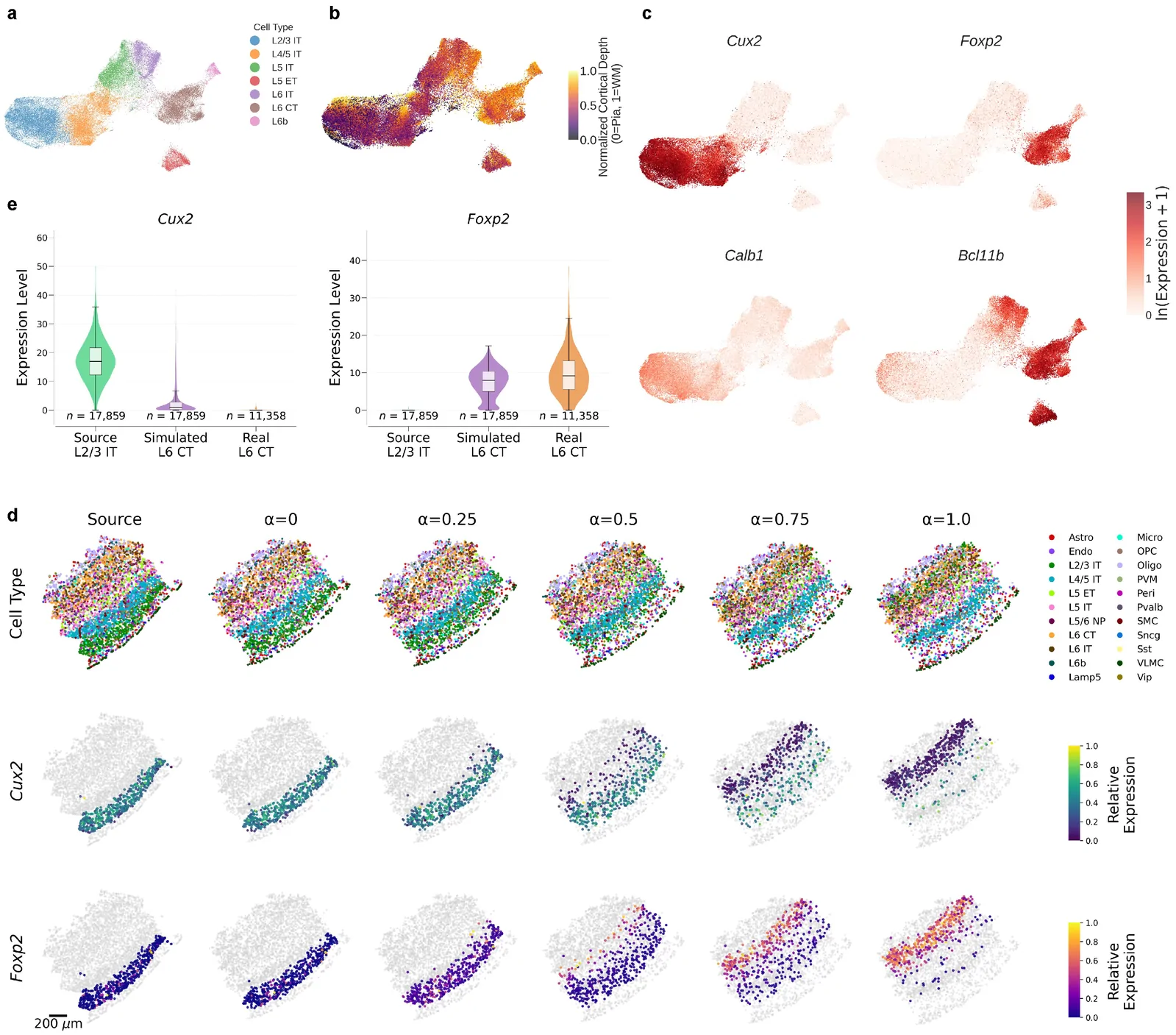
**Latent space topology and in silico reprogramming of the mouse cortex.** (**a**) UMAP projection of node-level latent representations for excitatory neurons in the MERFISH test set. Points denote single cells colored by annotated type. (**b**) UMAP embedding colored by unified normalized cortical depth (0 = pial surface, 1 = white matter, derived from geometry-corrected physical *y*-coordinates). (**c**) Expression distribution of superficial (*Cux2*, *Calb1*) and deep (*Foxp2*, *Bcl11b*) layer marker genes on the latent manifold. Color intensity reflects ln(x+1) -transformed expression, thresholded at the 99th percentile. (**d**) Continuous counterfactual intervention on a representative MERFISH cortical slice. Latent representations of L2/3 IT neurons were linearly shifted towards the L6 CT phenotype. Top: spatial map of cell types demonstrating progressive deep-layer migration of the reprogrammed population (green). Middle and bottom: spatial expression of *Cux2* and *Foxp2*. Target populations are colored by expression; others are gray. Columns denote increasing perturbation intensity ( α=0 to 1). Scale bar, 200 μm. (**e**) Quantification of reprogramming efficacy. *Cux2* and *Foxp2* expression across source L2/3 IT (*n* = 17,859), simulated L6 CT ( α=1.0 ), and real L6 CT neurons (*n* = 11,358). Box limits denote interquartile range (IQR), center lines denote median, and whiskers extend to 1.5× IQR.

Utilizing the highly multiplexed MERFISH mouse primary motor cortex atlas [18], we evaluated the capacity of the node encoder to parse complex tissue architecture. Although the model received no explicit anatomical supervision during training, the latent representations of excitatory neurons formed a structured, continuous manifold in the Uniform Manifold Approximation and Projection (UMAP) (Figure 2a). This manifold topology not only accurately recapitulated the anatomical laminar order from superficial (L2/3) to deep (L6) layers but also exhibited topological correspondence with the unified cortical depth of the tissue (Figure 2b).

Notably, this latent space encoded molecular identity and spatial context within a shared manifold while preserving their separability. Within the L5 cortical region, for instance, L5 extratelencephalic projecting (L5 ET) and L5 intratelencephalic projecting (L5 IT) neurons are highly intermixed in physical space. However, within the latent manifold, L5 ET neurons segregated into a distinct branch independent of the primary developmental trajectory. This topological separation was corroborated at the molecular level: the opposite poles of the manifold were robustly enriched for superficial (*Cux2*, *Calb1*) and deep (*Foxp2*, *Bcl11b*) layer marker genes (Figure 2c).

Given the continuous nature of the latent manifold, we investigated the feasibility of executing targeted cell identity reprogramming in silico. We defined a biological concept vector connecting the superficial and deep cortex, expressed as $\Delta c={\bar{z}}_{L6\_\mathrm{CT}}-{\bar{z}}_{L2/3\_\mathrm{IT}}$ . By employing linear interpolation with a coefficient α∈[0,1] , we progressively injected this vector into the latent representations of L2/3 IT neurons from the independent test set, thereby simulating a hypothetical phenotypic transition toward the deep-layer cortical identity.

At zero intervention ( α=0 ), the model faithfully reconstructed each source tissue in both molecular profile and spatial coordinates (Figure S1).The resulting counterfactual spatial maps demonstrated the model’s capacity to capture the intrinsic coupling between cellular identity and spatial positioning (Figure 2d). As the intervention intensity α increased, the target cell population underwent a molecular phenotypic transition accompanied by a coordinated shift in their generated spatial coordinates. In the model output, cells originally placed in superficial layers were repositioned toward the deep-cortex region, so that the predicted coordinates matched the spatial arrangement characteristic of deep-layer neurons. We speculate that this behavior reflects the model having learned a joint distribution governed by intrinsic geometric constraints of the tissue. When cellular identity was shifted toward the L6 CT phenotype, the model generated a corresponding spatial configuration consistent with deep-layer organization, so that the predicted transcriptional state and spatial position changed together. A paired Wilcoxon signed-rank test confirmed that the simulated reprogramming significantly suppressed *Cux2* and induced *Foxp2* expression in L2/3 IT neurons (*n* = 17,859; p<0.0001 ; Figure 2e). The reprogrammed cells nonetheless retained a residual transcriptional difference from the real L6 CT cohort, consistent with an incomplete phenotypic transition rather than full identity conversion. We interpret this result as evidence that the model has learned the association between molecular identity and laminar position, not as a claim that mature L2/3 neurons could physically migrate or be reprogrammed into L6 neurons in vivo.

### 3.3. Counterfactual Modeling of Histological Grade Transition in Breast Cancer

**Figure 3:**
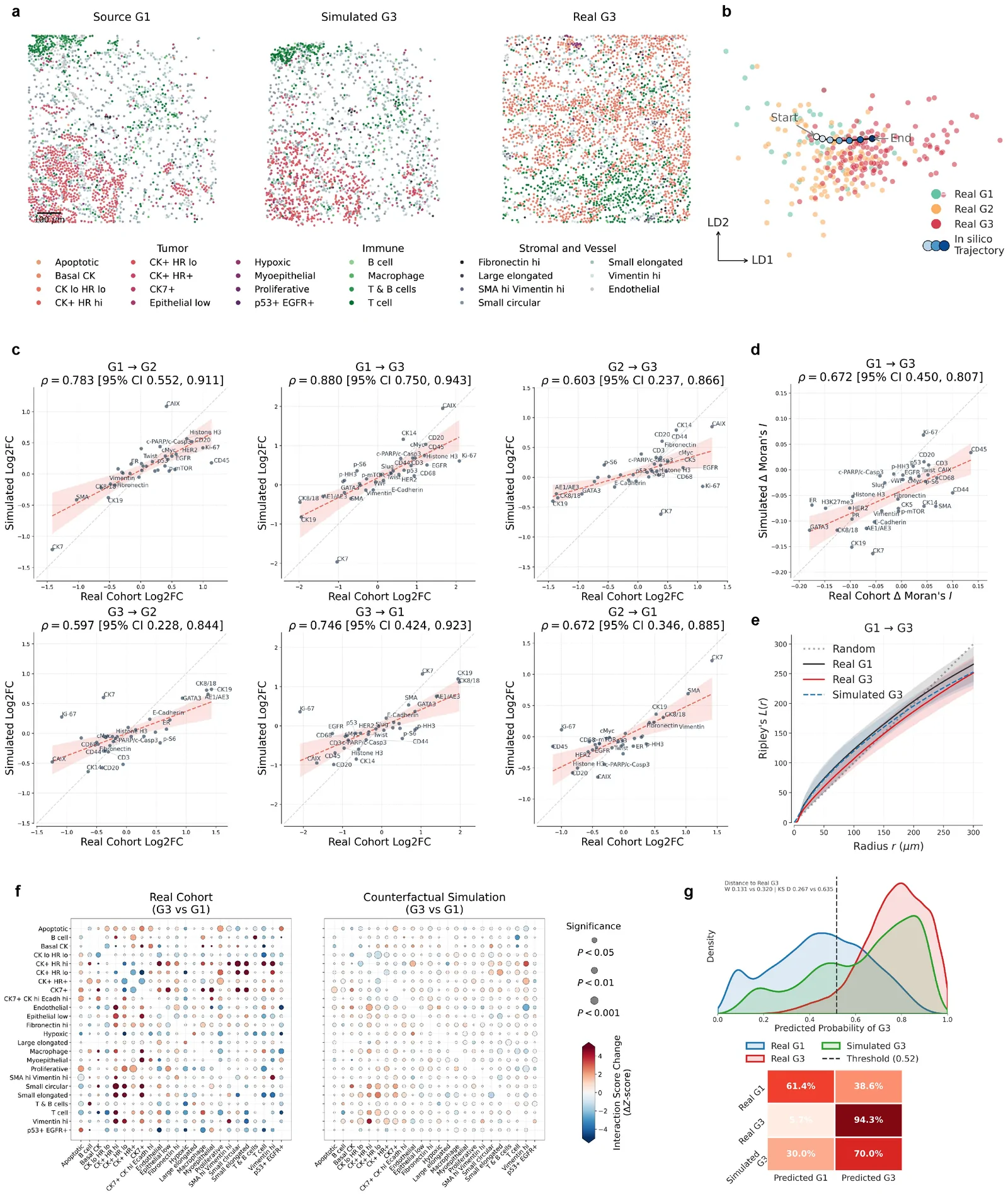
**In silico modeling of tumor grade transition and microenvironmental remodeling.** (**a**) Spatially resolved counterfactual simulation of histological grade transition. Comparison among a source Grade 1 (G1) tissue core (left), the corresponding simulated Grade 3 (G3) counterpart (middle), and an independent real G3 reference (right). Colors indicate cell lineages. Scale bar, 100 μm. (**b**) Linear discriminant analysis (LDA) projection of pseudo-bulk protein expression profiles from 289 tumor cores. Background points denote single tissue cores colored by histological grade. The in silico trajectory (blue points) tracks a representative test-set G1 core subjected to continuous counterfactual intervention targeting the G3 phenotype, with increasing intervention intensity ( α ranging from 0.25 to 1.5). (**c**) Evaluation of simulated expression dynamics across histological transitions. Scatter plots compare the real cohort $\log_{2}$ -transformed fold changes ( ${\mathrm{Log}}_{2}$ FC, *x*-axis) against simulated ${\mathrm{Log}}_{2}$ FC (*y*-axis) for ascending (top) and descending (bottom). Each point represents a protein marker ( n=33 ). Red dashed lines indicate linear regression fits with 95% confidence intervals (shaded); gray dashed lines indicate identity ( y=x ). Spearman’s rank correlation coefficients ( ρ ) with bootstrap 95% confidence intervals (1000 marker resamples) are shown. (**d**) Concordance of spatial autocorrelation shifts ( Δ Moran’s *I*) for the G1 → G3 transition. The real cohort Δ Moran’s *I* (*x*-axis, derived from real G3 vs. real G1) is compared against the simulated Δ Moran’s *I* (*y*-axis, derived from simulated G3 vs. source G1). (**e**) Tumor cell spatial distribution patterns evaluated via Besag’s transformation of Ripley’s *K*-function ( L(r)−r ) against spatial radius *r*. Curves represent the cohort-level mean spatial clustering intensity: real G1 source (black solid), real G3 target (red solid), simulated G3 (blue dashed), and complete spatial randomness (CSR; gray dotted). Shaded bands represent ± s.d. (**f**) Differential neighborhood enrichment dot plots capturing topological rewiring. Panels compare pairwise cell-cell interaction shifts ( ΔZ -score) within the real clinical cohort (left, real G3 vs. real G1) and the counterfactual simulation (right, simulated G3 vs. source G1). Dot size denotes statistical significance (two-sided independent Welch’s *t*-test for the real cohort; two-sided paired *t*-test for the simulation). (**g**) Quantitative assessment of phenotypic transition via a classifier-based realism assessment. Top: probability density of malignancy scores predicted by an independent random forest classifier trained on single-cell proteomic and local environmental features. The simulated G3 population (green) shifts substantially from the real G1 source (blue) toward the real G3 distribution (red), crossing the decision threshold (*p* = 0.52, dashed line). Inset text reports the distance from the simulated distribution to real G3 versus the real-G1-to-real-G3 distance (Wasserstein and Kolmogorov–Smirnov *D*) Bottom: corresponding row-normalized confusion matrix quantifying the classification conversion rates.

We next performed counterfactual simulations to model the transcriptional and spatial differences associated with histological grade in breast cancer. Using a grade vector defined in the node latent space, we simulated a distributional shift from a well-differentiated (Grade 1) toward a poorly differentiated (Grade 3) phenotype. Because histological grades are cross-sectional pathological categories rather than stages of a single temporal trajectory, we treat these simulations as transitions between phenotype distributions and not as a model of grade progression over time. Visually, the model-generated counterfactuals successfully transformed the architectural characteristics of Grade 1 tumor nests into the disorganized patterns typical of Grade 3 malignancies, while preserving the underlying tissue context of the source sample (Figure 3a). All grade-transition simulations were performed on the held-out test cohort. Projecting these trajectories onto a linear discriminant analysis (LDA) embedding of the patient cohort revealed that the continuous injection of the grade transition vector ( α∈[0.25,1.5] ) drove the latent representations of Grade 1 samples along a smooth manifold bridging the distinct clusters of low- and high-grade tumors (Figure 3b). The LDA axes were fitted on core-level pseudo-bulk profiles of real Grade 1–3 cores from the full Basel cohort; counterfactual trajectories for a held-out test core are projected onto these fixed axes without contributing to the fit. This confirms that SPAD-CFR has learned a continuous and navigable topological manifold spanning histological grades rather than merely memorizing discrete class-specific distributions.

To quantify molecular fidelity, we compared simulated differential expression profiles against real cohort-level grade-associated expression changes. The model demonstrated high predictive accuracy across all bidirectional grade transitions, in both the ascending (Grade 1 to 2, Grade 1 to 3, and Grade 2 to 3) and the corresponding descending directions. As shown in Figure 3c, the simulated $\log_{2}$ -transformed fold changes of 33 protein markers exhibited strong concordance with real cohort-level grade-associated expression changes (e.g., Spearman’s ρ=0.880 for Grade 1 to 3; p<0.001 ), consistent across all six bidirectional grade transitions.

Beyond molecular fidelity, SPAD-CFR also captured the complex spatial reorganization associated with histological grade transition. The simulated shifts in spatial autocorrelation ( Δ Moran’s *I*) for the Grade 1 to 3 transition showed a significant positive correlation with real cohort-level observations (Figure 3d), indicating accurate inference of marker-specific spatial patterning. Consistently, SPAD-CFR recapitulated the spatial dispersal pattern associated with higher-grade tumors, with counterfactual spatial distribution curves shifting from the Grade 1 baseline towards the Grade 3 profile (Figure 3e). These spatial changes were further accompanied by coordinated shifts in cell-cell interaction patterns within the tumor microenvironment, with the differential neighborhood enrichment profiles of counterfactual simulations closely mirroring those of the real cohort (Figure 3f).

Finally, we assessed the realism of the generated profiles with a classifier-based test using an independent random forest classifier [31] trained to distinguish histological grade (Grade 1 vs. Grade 3) based on single-cell proteomics and local neighborhood statistics. When presented with counterfactual profiles generated from Grade 1 inputs, the classifier assigned Grade 3 malignancy probabilities comparable to real Grade 3 cells, achieving a conversion rate of 70.0% (Figure 3g). Collectively, these results demonstrate that SPAD-CFR generates counterfactuals with authentic multidimensional grade-associated signatures, spanning molecular expression, spatial topology, and microenvironmental context.

### 3.4. Subtype-Specific Spatial Remodeling at the Invasive Tumor Margin

**Figure 4:**
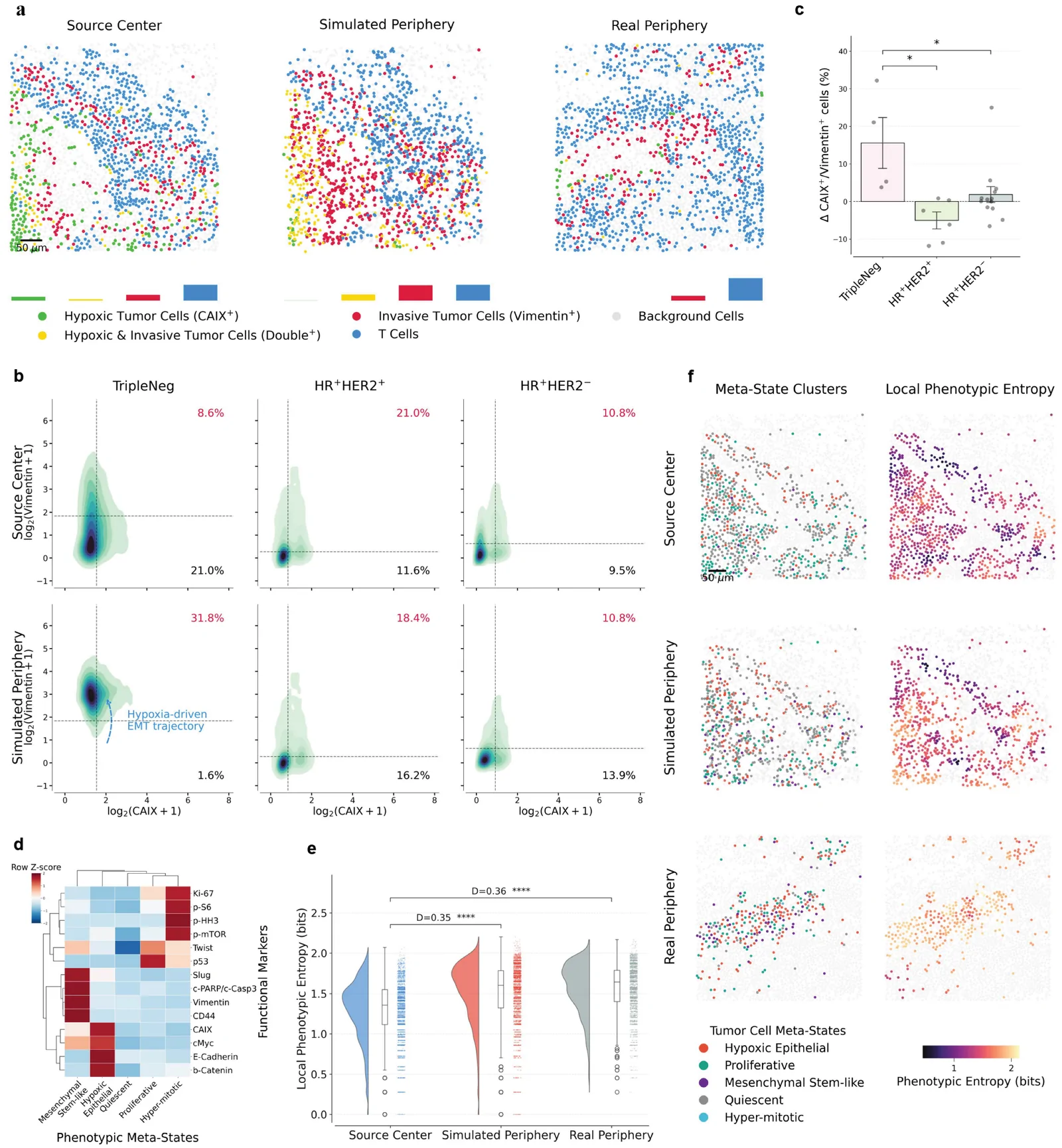
**Subtype-specific microenvironmental reprogramming and spatial entropy in breast cancer.** (**a**) Spatial maps comparing a source central core (left), the simulated peripheral state (middle), and a patient-matched real peripheral core (right) in triple-negative breast cancer (TNBC). Points denote distinct cell lineages: hypoxic tumor cells ( ${\mathrm{CAIX}}^{+}$ , green), invasive tumor cells ( ${\mathrm{Vimentin}}^{+}$ , red), double-positive clones ( ${\mathrm{CAIX}}^{+}{\mathrm{/Vimentin}}^{+}$ , yellow), and T cells (blue). Positivity thresholds correspond to the ≥60th percentile of expression across pooled real TNBC tumor cells in the test set. Bottom bars quantify absolute cell counts. Scale bars, 50 μm. (**b**) 2D kernel density estimation of single-cell state transitions. Contour plots display the joint distribution of $\log_{2}\left(x+1\right)$ -transformed CAIX and Vimentin expression for tumor cells in the source center (top) and simulated periphery (bottom) across clinical subtypes. Dashed lines denote positivity thresholds (≥60th percentile derived independently from real tumor cells per subtype). Percentages indicate the fraction of cells per quadrant. The blue arrow highlights a putative hypoxia-driven epithelial–mesenchymal transition (EMT) trajectory in TNBC. (**c**) Subtype-specific spatial plasticity. Absolute change ( Δ ) in the percentage of ${\mathrm{CAIX}}^{+}{\mathrm{/Vimentin}}^{+}$ double-positive tumor cells following simulated central-to-peripheral transition. Bars represent mean ± s.e.m., with dots denoting individual tumor cores. Significance was determined via the Kruskal–Wallis test with Benjamini–Hochberg FDR correction (* p<0.05 ). (**d**) Identification of intra-tumoral phenotypic meta-states. Hierarchically clustered heatmap of mean expression profiles for 14 functional markers across five tumor cell meta-states, identified via *K*-means clustering in the TNBC cohort. The color scale indicates row-standardized expression (*Z*-score). (**e**) Quantification of local phenotypic entropy for tumor cells in TNBC cohorts. Entropy values reflect the spatial diversity of functional meta-states within each cell’s 20 nearest neighbors ( k=20 ). Raincloud plots display kernel density estimations, individual cells, and box plots (center line, median; box limits, interquartile range (IQR); whiskers, 1.5× IQR). Significance of distributional shifts was assessed via two-sided Kolmogorov–Smirnov tests (*D*, test statistic; **** p<0.0001 ). (**f**) Spatial mapping of phenotypic remodeling and microenvironmental entropy. Tissue coordinate maps comparing the source center (top), simulated periphery (middle), and patient-matched real periphery (bottom). Left: tumor cells colored by discrete functional meta-states (defined in **d**). Right: corresponding topological maps of local phenotypic entropy (bits). Scale bars, 50 μm.

To examine whether SPAD-CFR could disentangle intrinsic cellular states from spatial microenvironmental constraints, we applied the framework to model the center-to-periphery spatial transition axis in breast cancer. Conventional paradigms often characterize the invasive tumor margin as a uniformly well-oxygenated front; however, highly aggressive subtypes such as Triple-Negative Breast Cancer (TNBC) frequently outgrow their vascular supply, generating severe marginal hypoxia [34]. By isolating a spatial transition vector ( $\Delta c_{\mathrm{loc}}$ ) from the node latent space and applying it to central TNBC cores, SPAD-CFR challenged this simplified re-oxygenation model. Rather than predicting mutually exclusive expression of hypoxic and invasive markers, the counterfactual simulation generated a concomitant upregulation of Carbonic Anhydrase IX (CAIX) and Vimentin (Figure 4a). Single-cell state transition mapping quantitatively corroborated this modeled trajectory, revealing a shift of the tumor population into a double-positive state (from 8.6% to 31.8%), consistent with the established mechanism of hypoxia-driven Epithelial–Mesenchymal Transition (EMT) at the invasive front [35,36] (Figure 4b). This remodeling was subtype-specific, with TNBC cores showing markedly greater induction of double-positive clones compared to luminal subtypes ( ${\mathrm{HR}}^{+}{\mathrm{HER2}}^{+}$ and ${\mathrm{HR}}^{+}{\mathrm{HER2}}^{-}$ ), which remained comparatively refractory to this reprogramming ( p<0.05 , Kruskal–Wallis test with Benjamini–Hochberg correction; Figure 4c).

Invasive margins are further characterized by pronounced intra-tumoral heterogeneity (ITH) arising from the coexistence of functionally distinct subclonal populations [37]. To assess whether SPAD-CFR could reconstruct this higher-order ecological complexity, tumor cells from the TNBC cohort were partitioned into five discrete phenotypic meta-states, ranging from quiescent and hypoxic baselines to mesenchymal stem-like and hyper-mitotic functional modules (Figure 4d). Local phenotypic entropy was then computed within each cell’s spatial neighborhood as a measure of microenvironmental diversity (Figure 4e). The counterfactual center-to-periphery transition drove a statistically significant distributional shift towards a higher-entropy, more admixed microenvironmental state (Kolmogorov–Smirnov test, D=0.35 , p<0.0001 ), dismantling the low-entropy homotypic clusters characteristic of the source central core. Spatial topological mapping visually corroborated this systemic elevation in local entropy (Figure 4f), with the localized emergence and physical intermingling of mesenchymal and hyper-mitotic subclones closely recapitulating the architectural organization observed in a representative real peripheral core from the same patient. Collectively, these results demonstrate that SPAD-CFR can simulate spatially anchored, non-linear microenvironmental remodeling and multicellular ecosystem restructuring in a subtype-specific manner.

### 3.5. In Silico Immune Checkpoint Blockade Reveals Spatially Anchored Cytotoxicity and Adaptive Resistance

**Figure 5:**
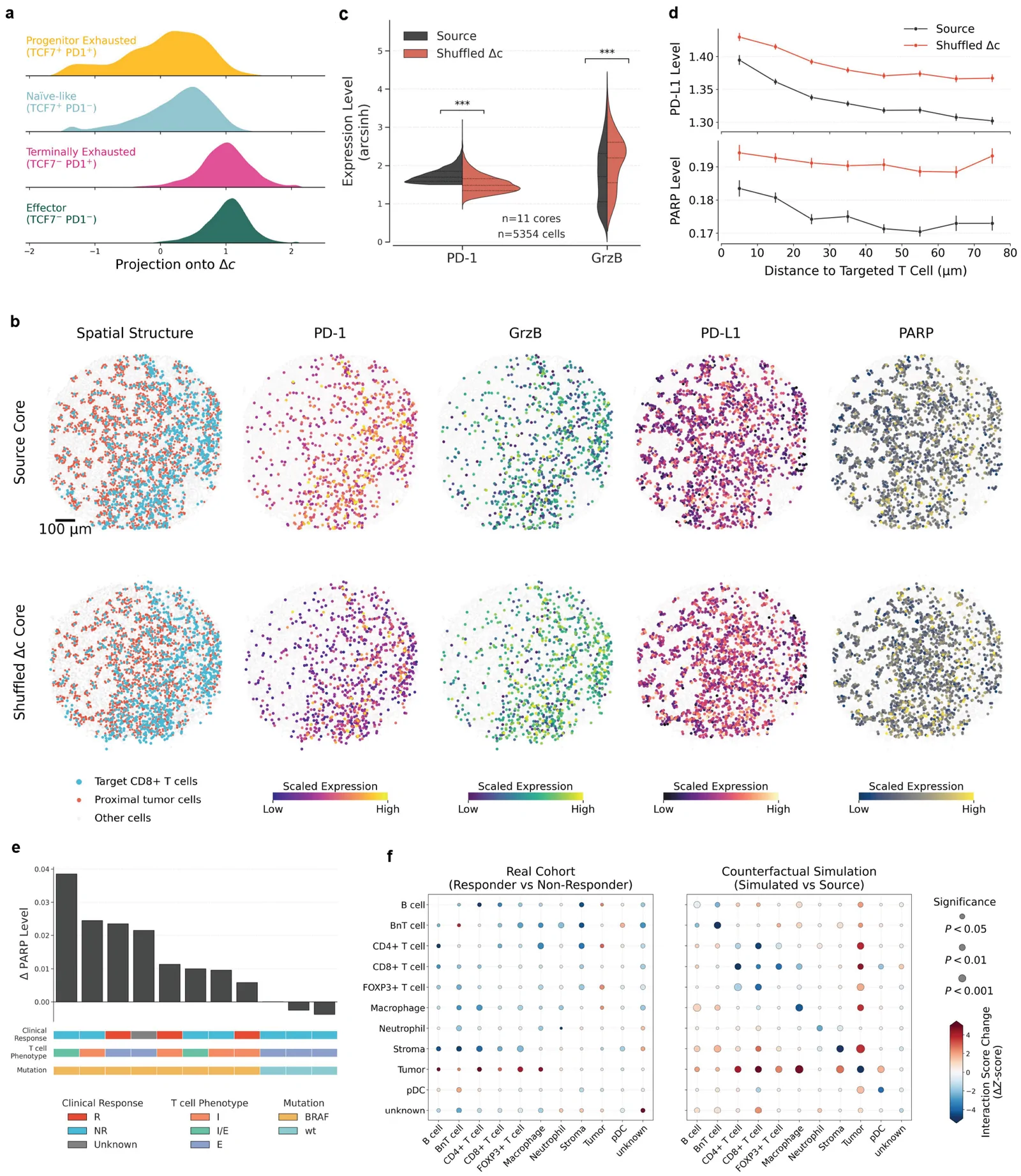
In silico targeted immune checkpoint blockade decodes spatial cytotoxicity and adaptive resistance. (**a**) Latent projection of ${\mathrm{CD8}}^{+}$ T cell functional trajectories. Ridge plots display density distributions of four distinct ${\mathrm{CD8}}^{+}$ T cell subsets projected onto the in silico activation axis ( Δc ), where Δc denotes the in silico activation vector derived from the node latent space. (**b**) Spatial mapping of in silico targeted immune checkpoint blockade (ICB). Spatial maps of a representative melanoma core before (source, top) and after (simulated, bottom) node-level perturbation. The spatial structure map (left) highlights target ${\mathrm{PD-1}}^{+}{\mathrm{CD8}}^{+}$ T cells (blue) and proximal tumor cells within a 20-μm radius (red). Accompanying marker maps demonstrate on-target phenotypic shifts (PD-1, GrzB) and indirect bystander responses (cleaved PARP, PD-L1). Scale bars, 100 μm. (**c**) On-target phenotypic reversal of exhausted ${\mathrm{CD8}}^{+}$ T cells. Split violin plots of arcsinh-transformed PD-1 and GrzB expression within targeted ${\mathrm{CD8}}^{+}$ T cells (*n* = 5354 targeted cells pooled from the test cohort, restricted to cores with >30 targets). Distributions compare the unperturbed baseline (source, dark grey) and generative predictions (simulated, red). Dashed lines denote quartiles and median. Significance was determined via a two-sided paired Wilcoxon signed-rank test computed at the core level ( n=11 cores, *** *p* < 0.001). (**d**) Spatial distance-decay of simulated bystander responses at cohort scale. Line plots quantify arcsinh-transformed expression of PD-L1 (top) and PARP (bottom) in melanoma cells as a function of physical distance to the nearest targeted ${\mathrm{CD8}}^{+}$ T cell. Data are aggregated into 10-μm spatial bins (0–80 μm) across the filtered test cohort (same inclusion criteria as **c**). Points and error bars represent the mean ± s.e.m. for the source baseline (dark grey) and simulated state (red). (**e**) Inter-core heterogeneity of simulated bystander cytotoxic responses. Waterfall plot of individualized shifts in bystander cytotoxicity ( Δ PARP, simulated minus source) across independent test cores. Δ PARP was calculated exclusively for proximal melanoma cells within a 20-μm radius of perturbed T cells. Annotation tracks denote clinical response (R, responder; NR, non-responder; Unknown), baseline spatial infiltration phenotype (I, inflamed; I/E, mixed; E, excluded), and *BRAF* genotype (mutant vs. wt). (**f**) Differential neighborhood enrichment capturing microenvironmental rewiring. Dot plots compare pairwise cell-cell interaction shifts ( ΔZ -score) between the real cohort (left, responder vs. non-responder) and the in silico simulation (right, simulated post-activation vs. source pre-intervention). Color denotes interaction shift magnitude (red, increased proximity/mixing; blue, segregation/exclusion). Dot size reflects statistical significance (two-sided independent Welch’s *t*-test for the real cohort; two-sided paired *t*-test for the simulation).

We next applied SPAD-CFR to model an in silico activation intervention on exhausted ${\mathrm{CD8}}^{+}$ T cells in a multiplexed melanoma cohort [20], as a latent approximation of the T-cell reinvigoration associated with immune checkpoint blockade (ICB). An ICB transition vector ( Δc ) was derived within the node latent space as the difference between the centroid of ${\mathrm{GrzB}}^{+}$ cells and that of the progenitor exhausted subpopulation, rather than the nominal ${\mathrm{TCF}7}^{-}{\mathrm{PD}-1}^{-}$ subset, because Granzyme B is the canonical marker of cytotoxic effector ${\mathrm{CD8}}^{+}$ T cells, whereas the ${\mathrm{TCF}7}^{-}{\mathrm{PD}-1}^{-}$ definition does not itself index cytotoxic capacity. Projecting the four ${\mathrm{CD8}}^{+}$ T cell subsets onto this axis revealed a coherent separation along the activation–exhaustion continuum (Figure 5a), suggesting that the node encoder captures functionally meaningful structure within the T cell state-space [38].

We then applied the Δc vector selectively to the ${\mathrm{PD-1}}^{+}{\mathrm{CD8}}^{+}$ T-cell population. Because PD-1 marks the cells engaged by anti-PD-1 antibodies [39], this perturbation induced on-target phenotypic shifts characterized by PD-1 downregulation and concomitant GrzB upregulation, both in representative spatial maps (Figure 5b) and across the pooled test cohort ( n=5354 targeted cells, p<0.0001 ; Figure 5c). This localized reinvigoration further drove spatially restricted bystander responses within the unperturbed tumor compartment (Figure 5b). Cohort-scale quantification revealed that the induction of tumor apoptosis (cleaved PARP) and adaptive immune resistance (PD-L1) both followed distance-dependent decay gradients radiating from the nearest targeted T cells (Figure 5d), consistent with the biophysical constraints of contact-dependent cytotoxicity and diffusion-limited cytokine signaling, including IFN- γ -mediated PD-L1 upregulation [40].

To evaluate inter-core heterogeneity in simulated therapeutic response, we quantified individualized bystander cytotoxicity ( ΔPARP ) for proximal melanoma cells across independent test cores (Figure 5e). All clinical responders exhibited positive apoptotic induction in silico, whereas cores refractory to the simulated intervention ( ΔPARP<0 ) were exclusively characterized by an immune-excluded baseline spatial topology [41]. This indicates that SPAD-CFR recognizes physical stromal barriers that preclude direct T cell–tumor engagement, gating simulated therapeutic efficacy through individual spatial architectures.

Finally, we contrasted real cohort-level clinical baselines with counterfactual simulations to assess microenvironmental rewiring at the systems level (Figure 5f). Clinical responders exhibited elevated baseline heterotypic tumor–immune mixing indicative of pre-existing immunity, a pattern that SPAD-CFR reproduced by shifting the simulated post-intervention state toward an inflamed topology. Furthermore, whereas the real cohort baseline maintained intact homotypic tumor–tumor interactions, the counterfactual simulation predicted a disruption of homotypic tumor clustering as a consequence of spatially executed T cell cytotoxicity, indicating that SPAD-CFR models structural remodeling of the tumor architecture rather than merely overlaying expression changes onto a fixed spatial grid.

## 4. Discussion

The transition from purely observational spatial omics to causal, predictive modeling remains a central challenge in systems biology. While recent advances in machine learning have enabled sophisticated single-cell perturbation predictions [7,8], extending these frameworks to encompass the physical topology and spatially anchored paracrine signaling of intact tissues has remained a substantial challenge. Here, we introduced SPAD-CFR, a generative framework that unites structural causal modeling with point cloud diffusion to execute in silico targeted microenvironmental reprogramming. By demonstrating its capacity to infer anatomical cortical trajectories, simulate subtype-specific hypoxia-driven invasion, and characterize the spatially restricted consequences of an activation intervention on exhausted T cells, we established a computational testbed for spatially resolved counterfactual inference.

A key methodological distinction of SPAD-CFR lies in its departure from fixed-grid spatial networks and virtual-cell abstraction. Existing spatial generative frameworks predominantly rely on deterministic graph neural networks (GNNs) or convolutional architectures that propagate perturbation effects across static spatial topologies [13,15]. However, therapeutic interventions and disease-associated state transitions rarely alter transcriptomic profiles without concomitant remodeling of physical architecture. By projecting the tissue as a continuous multidimensional point cloud and independently corrupting both molecular features and physical coordinates within a joint diffusion process, SPAD-CFR decouples intrinsic cellular identity from extrinsic spatial geometry.

As demonstrated in our melanoma in silico ICB simulations, the model did not merely generate a uniform upregulation of cytotoxic effectors; it inferred distance-decay gradients of bystander apoptosis and adaptive immune resistance (PD-L1) in neighboring tumor cells. More importantly, it predicted therapeutic failure in tumor cores characterized by immune-excluded topologies, consistent with established patterns of clinical resistance [41]. This capability to execute localized perturbations and read out systemic structural rewiring enables researchers to computationally screen hypothetical drug targets, identify physical stromal barriers, and evaluate patient-specific therapeutic vulnerabilities prior to costly clinical trials or in vivo spatial perturbation experiments. In practice, a single counterfactual-style generation is fast and memory-light, remaining well within routine GPU budgets at the tissue sizes analyzed (Figure S2).

It is crucial to clarify what our validation results establish. The comparative results we report indicate that the generated tissue samples closely resemble the naturally occurring target population, including high-grade tumors, peripheral tumor margins, and the microenvironment of clinically responsive melanomas. These are population-level findings that demonstrate the model’s ability to reproduce population-level associations and map the source samples to the target distribution. However, differences between cross-sectional patient cohorts do not equate to the changes that occur when actual tissues are perturbed, as patient-specific genotypes, subtypes, treatment histories, and other factors can all contribute to between-group differences. Direct validation of the predicted effects of real interventions on specific tissues would require spatial perturbation data, longitudinal data, or matched pre- and post-treatment data, none of which are available in the cohort analyzed in this study. Therefore, we primarily present the current results as simulation findings that raise hypotheses, and we consider perturbation validation to be an important area for future work.

Despite these results, we acknowledge several methodological and biological limitations. First, our structural concept vectors ( Δc ) are extracted via centroid differences, which assumes a locally linear Euclidean manifold within the node latent space. Although biological differentiation and pathological state transitions frequently follow non-linear trajectories, our empirical results suggest that the non-linear attention layers of the generative reverse process can decode these linear interventions into non-linear transcriptomic and spatial dynamics. Second, our Structural Causal Model approximates the exogenous spatial noise ( ϵ ) as conditionally independent from the target cell states via the isotropic Gaussian assumption of diffusion models. In highly complex tissues, unobserved confounders may couple intrinsic cell states with geometric noise; extracellular matrix (ECM) stiffness and localized chemokine gradients represent notable examples of such dependencies, which are not explicitly resolved by the current SCM. Finally, while SPAD-CFR infers spatial topology from data distributions, it lacks explicit biophysical constraints, such as cell volume exclusion or mechanical tension, which govern tissue morphogenesis. Throughout, the number of cells in each tissue is held fixed. Processes that add or remove cells are outside our current scope.

Future iterations of this framework could integrate non-linear manifold learning and generalized causal discovery algorithms to refine the perturbation axes. Additionally, incorporating physical inductive biases and expanding the model to encompass 3D spatial transcriptomics will further enhance its microenvironmental resolution. Systematic comparison against non-spatial and graph-based generative baselines, together with component-level ablations (coordinate branch, pairwise-distance loss, node-level conditioning, linear attention) and multi-seed confidence intervals, likewise remains important future work. We also plan to test a held-out-marker approach in future work. In this design, the intervention vector is built using one set of markers and then evaluated on a completely separate set. This would be the most rigorous way to assess whether the model truly extrapolates beyond the input markers. Ultimately, SPAD-CFR provides a mathematical framework for executing in silico spatial experiments, advancing the use of in silico spatial experiments for hypothesis generation in oncology and precision medicine.

## 5. Conclusions

In this study, we developed SPAD-CFR, a structurally causal diffusion framework designed to execute spatially targeted counterfactual reprogramming at single-cell resolution. By treating tissue architectures as continuous point clouds and independently modeling molecular features and physical coordinates, our approach addresses key limitations of existing non-spatial and static-graph virtual tissue models. We demonstrated that SPAD-CFR captures the dynamic, contact-dependent remodeling of tissue microenvironments. It reconstructed anatomical cortical trajectories, decoded subtype-specific hypoxia-driven spatial plasticity at invasive tumor margins, and simulated the emergent bystander cytotoxicity and homotypic nest disassembly induced by a targeted activation intervention that models checkpoint blockade.

Ultimately, SPAD-CFR establishes a computational testbed for spatial counterfactual inference. By decoupling intrinsic cellular states from extrinsic geometric constraints, this framework enables researchers to computationally screen therapeutic perturbations, anticipate microenvironmental rewiring, and identify physical mechanisms of drug resistance. As spatial multi-omics technologies continue to scale, generative causal frameworks like SPAD-CFR will be instrumental in accelerating the transition from observational tissue mapping to the generation of testable, spatially resolved hypotheses for precision oncology.

## Data Availability

All datasets analyzed in this study are publicly available from their original publications. The MERFISH mouse primary motor cortex dataset [18] is accessible via the Brain Image Library at https://doi.org/10.35077/g.21. The IMC breast cancer datasets (Basel and Zurich cohorts) [19] are available on Zenodo at https://doi.org/10.5281/zenodo.3518284. The multiplexed melanoma IMC dataset [20] is available on Zenodo, with raw single-cell expression data at https://doi.org/10.5281/zenodo.6004986, and processed data at https://doi.org/10.5281/zenodo.5994136. The source code, configuration files, and preprocessing scripts are available at https://github.com/WenhuiDing/SPAD-CFR (accessed on 5 July 2026). The processed training and test datasets and the trained model checkpoints are permanently archived on Zenodo (DOI: https://doi.org/10.5281/zenodo.20254865).

## Supplementary Materials

The following supporting information can be downloaded at: https://www.mdpi.com/article/10.3390/biology15141097/s1, Figure S1: α=0 reconstruction benchmark across the four datasets; Figure S2: Per-sample inference benchmark on the MERFISH mouse cortex test set; Figure S3: Ablation of DDIM inversion in the MERFISH mouse cortex; Figure S4: Concept-vector specificity controls in the IMC melanoma cohort; Table S1: Implementation and training details of SPAD-CFR.
