# Correction: Ding et al. Counterfactual Diffusion Modeling Enables Spatially Targeted Reprogramming of Tissue Microenvironments. *Biology* 2026, *15*, 1097

**DOI:** 10.3390/biology15161380

**Published:** 2026-08-13

**Authors:** Wenhui Ding, Zhenhua Luo, Yuanyan Xiong

**Affiliations:** 1 Department of Pediatric Surgery, The First Affiliated Hospital, Sun Yat-sen University, Guangzhou 510080, China; dingwh6@mail2.sysu.edu.cn; 2 Institute of Precision Medicine, The First Affiliated Hospital, Sun Yat-sen University, Guangzhou 510080, China; 3 Key Laboratory of Gene Engineering of the Ministry of Education, Institute of Healthy Aging Research, School of Life Sciences, Sun Yat-sen University, Guangzhou 510275, China

In the published publication [1], there was an error regarding the affiliations for Wenhui Ding and Zhenhua Luo. In addition to affiliation 1, the updated affiliations should include: 1. Department of Pediatric Surgery, The First Affiliated Hospital, Sun Yat-sen University, Guangzhou 510080, China; 2. Institute of Precision Medicine, The First Affiliated Hospital, Sun Yat-sen University, Guangzhou 510080, China. The authors state that the scientific conclusions are unaffected. This correction was approved by the Academic Editor. The original publication has also been updated.
