# Supplementary material for: Counterfactual Diffusion Modeling Enables Spatially Targeted Reprogramming of Tissue Microenvironments
Source: Biology (Basel). 2026 Jul 8;15(14):1097. doi: 10.3390/biology15141097 (PMC13406000; doi:10.3390/biology15141097)
Supplement: Supplementary file 1 [file biology-15-01097-s001.zip › biology-4409703-supplementary.pdf]

Supplementary Materials

Counterfactual Diffusion Modeling Enables Spatially Targeted Reprogramming of Tissue Microenvironments

Wenhui Ding, Zhenhua Luo, and Yuanyan Xiong

Biology, MDPI

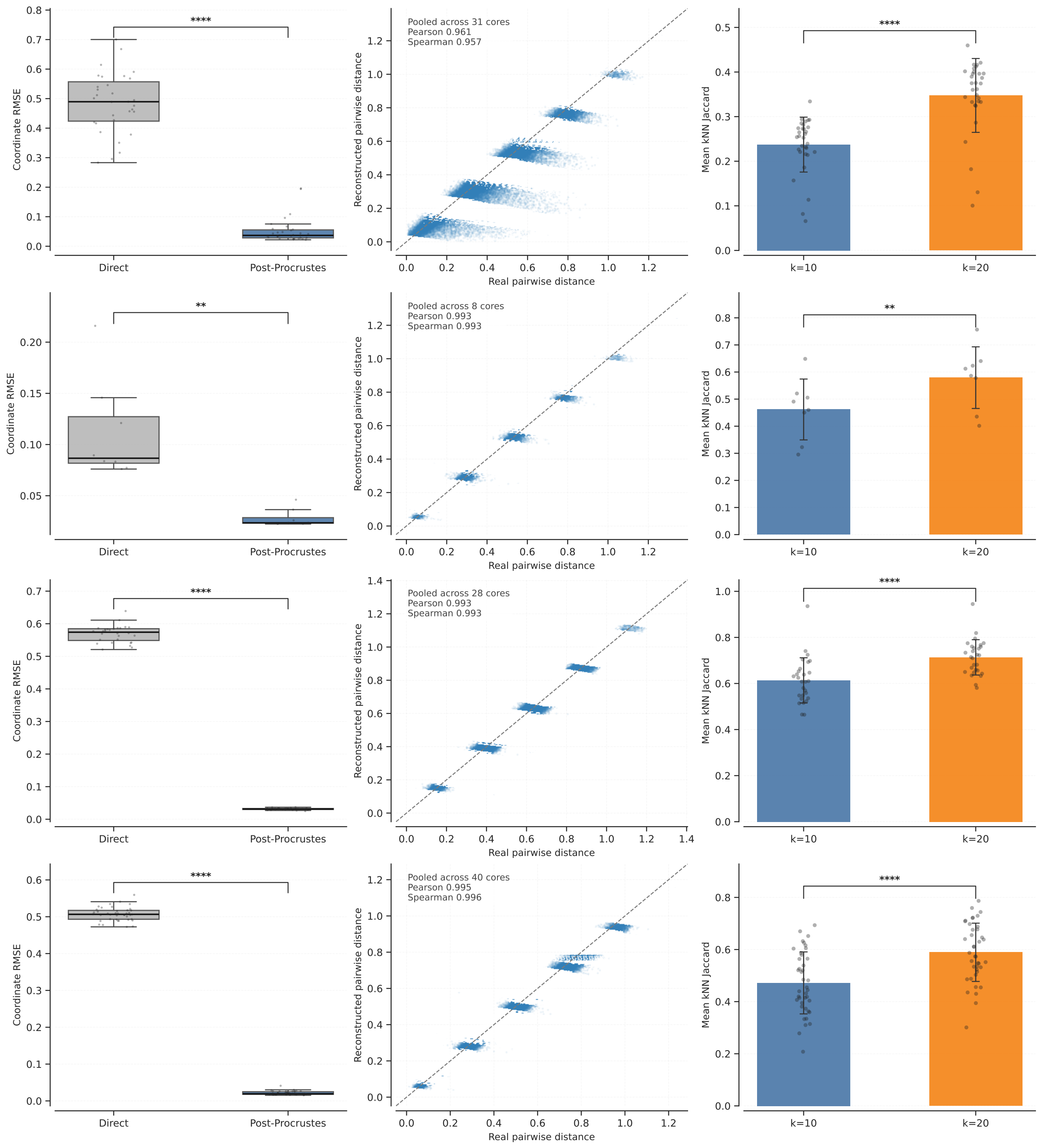

Figure S1: $\alpha = 0$ **reconstruction benchmark across the four datasets.** Rows correspond to MERFISH mouse cortex, IMC breast Basel cohort, IMC breast Zurich cohort, and IMC melanoma cohort. *Left column*, per-sample coordinate reconstruction error, comparing direct RMSE (no alignment) with post-Procrustes RMSE after rigid alignment to the source coordinates. *Middle column*, pairwise-distance fidelity between reconstructed and input coordinates, computed on cell pairs sampled and pooled across all samples of each dataset (up to ∼5,000 sampled pairs per sample); the grey dashed line denotes identity ($y = x$), and inset text reports the pooled Pearson and Spearman correlations. *Right column*, local neighborhood preservation, measured as the mean k-NN Jaccard overlap at $k = 10$ and $k = 20$ across samples. Boxplots summarize the cross-sample distribution (center line, median; box, IQR; whiskers, 1.5× IQR); bars indicate the mean and error bars denote SD across samples. Significance in the left and right columns was assessed by two-sided paired Wilcoxon signed-rank tests ($*P < 0.05$, $**P < 0.01$, $***P < 0.001$, $****P < 0.0001$; ns, not significant).

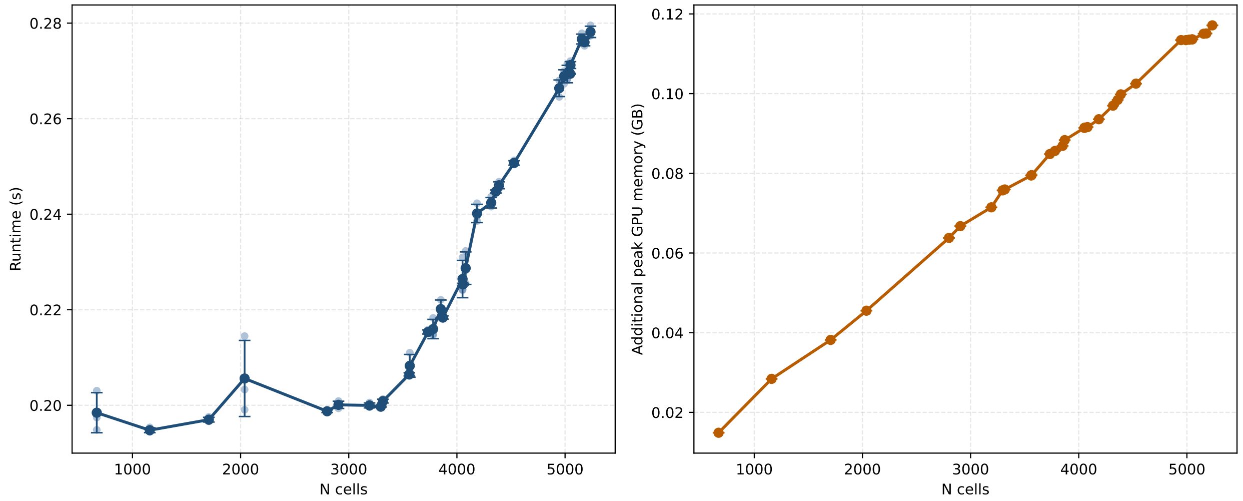

Figure S2: Per-sample inference benchmark on the MERFISH mouse cortex test set. Each point is one cortical slice, positioned by its true cell number N. *Left*, wall-clock runtime for a single counterfactual generation, measured after one-time model initialization and concept-direction (Δc) computation; runtime remains under 0.3 s across all slice sizes, staying roughly flat up to ∼3,000 cells and rising modestly thereafter. *Right*, additional peak GPU memory above the loaded-model baseline, which increases approximately linearly in N and stays under 0.12 GB up to ∼5,200 cells. Points and error bars denote mean ± SD across repeated runs.

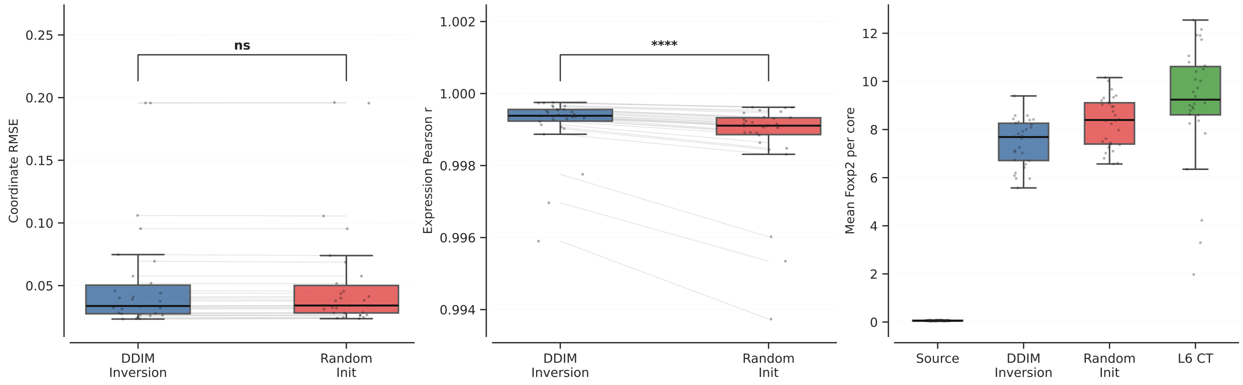

Figure S3: Ablation of DDIM inversion in the MERFISH mouse cortex. *Left*, non-target spatial context preservation, quantified by post-Procrustes coordinate RMSE. *Middle*, non-target expression preservation, quantified by the median per-cell Pearson correlation. *Right*, target phenotype attainment, quantified by mean *Foxp2* expression in target L2/3 IT cells relative to the source state and the reference L6 CT population. Comparison is between counterfactuals initialized by DDIM inversion and by random Gaussian noise, under identical conditioning and intervention. Dots indicate individual slices ($n = 31$) and boxes show the interquartile range with median. Significance was determined via two-sided paired Wilcoxon signed-rank tests in the left and middle panels ($^{*}P < 0.05$, $^{**}P < 0.01$, $^{***}P < 0.001$, $^{****}P < 0.0001$; ns, not significant).

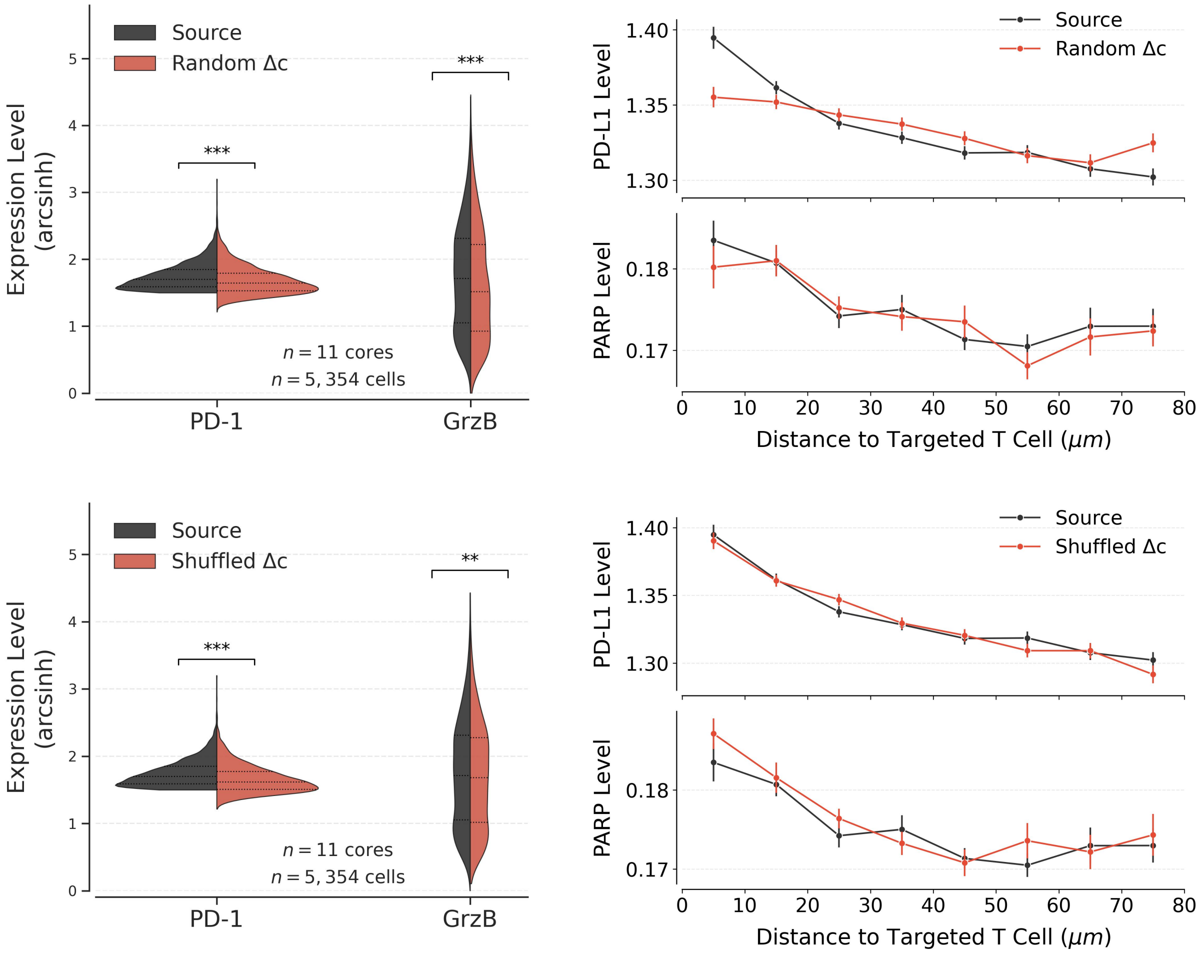

Figure S4: Concept-vector specificity controls in the IMC melanoma cohort. The biological activation vector Δc was replaced with two negative controls while holding the targeted PD-1^{+} CD8^{+} T cells and the full pipeline fixed, and the on-target and bystander analyses were re-run for each. *Top row*, norm-matched random Δc; *bottom row*, dimension-shuffled Δc. *Left panels*, on-target expression (arcsinh) of PD-1 and Granzyme B in targeted CD8^{+} T cells (n = 5,354 cells pooled from n = 11 cores with $>$ 30 targets each), comparing the unperturbed source (dark grey) with the control-perturbed prediction (red); dashed lines denote quartiles and median. *Right panels*, spatial distance-decay of bystander PD-L1 (top) and cleaved PARP (bottom) in melanoma cells as a function of distance to the nearest targeted T cell, binned in 10-μm intervals; points and error bars denote mean $\pm$ s.e.m. for the source (black) and control-perturbed (red) states. Neither control reproduces the on-target effector induction or a consistent bystander response. Significance for the on-target panels was assessed by two-sided paired Wilcoxon signed-rank tests (*$P < 0.05$, **$P < 0.01$, ***$P < 0.001$, ****$P < 0.0001$).

Table S1: Implementation and training details of SPAD-CFR.

Component	Setting
**Architecture**	
Encoder hidden dimension (feature / co-ordinate)	256 / 32
Latent dimension (d_{node})	128
Denoising transformer (layers / heads)	8 / 16
Encoder transformer (layers / heads)	3 / 16
Attention mechanism	Linear attention, $\mathcal{O}(N)$ complexity
Activation function	GELU
**Diffusion and sampling**	
Noise schedule	Cosine ($\nu = 2$)
Diffusion steps (T)	1000
DDIM inversion / sampling	100 effective steps (stride 10)
**Optimization**	
Optimizer	AdamW (AMSGrad), weight decay 10^{-12}
Learning rate	5×10^{-4}, constant
Batch size / epochs	6 / 1000
Loss weighting (position : feature)	10 : 1
Random seeds (training / inference)	0 / 42
Checkpoint selection	Fixed-interval saving (every 250 epochs); no validation-based selection
**Hardware and runtime**	
Hardware	1× NVIDIA RTX 4090 (24 GB)
Training time (cortex / Basel / Zurich / melanoma)	0.75 / 4.67 / 3.83 / 3.18 h
**Data handling**	
Expression values (IMC)	Raw values in the training path; arcsinh (cofactor 1) for downstream annotation only
Coordinate normalization	Per-section min–max to $[-0.5, 0.5]$, then mean-centering of valid cells
Variable cell number	Per-section graphs, ≤ 4096 cells each (chunked if larger); zero-padded and masked
**Downstream Random Forest classifier**	
Configuration	100 trees; max depth 15; min 5 samples/leaf; balanced class weights; OOB enabled
Feature vector	$3F$ per cell: own profile, k=10 neighbor mean, neighbor SD (Basel: $33 \times 3 = 99$)
Decision threshold	Youden's J on the training-set OOB ROC
